# Increased serum adipokines are associated with sarcopenia in non‐obese women with rheumatoid arthritis

**DOI:** 10.1002/kjm2.12823

**Published:** 2024-04-11

**Authors:** Tzu‐Jung Fang, Min‐Hsi Chiu, Ming‐Shyan Huang, Chia‐Yen Dai, Yao‐Tsung Yeh, Jeng‐Hsien Yen

**Affiliations:** ^1^ Graduate Institute of Clinical Medicine, College of Medicine Kaohsiung Medical University Taiwan; ^2^ Division of Geriatrics and Gerontology, Department of Internal Medicine Kaohsiung Medical University Hospital Taiwan; ^3^ School of Medicine, College of Medicine Kaohsiung Medical University Taiwan; ^4^ Aging and Disease Prevention Research Center Fooyin University Kaohsiung Taiwan; ^5^ Department of Medical Laboratory Sciences and Biotechnology Fooyin University Kaohsiung Taiwan; ^6^ E‐Da Cancer Hospital Kaohsiung Taiwan; ^7^ School of Medicine I‐Shou University Kaohsiung Taiwan; ^8^ Hepatobiliary Division, Department of Internal Medicine, Kaohsiung Medical University Hospital Kaohsiung Medical University Taiwan; ^9^ Division of Rheumatology, Allergy, and Immunology, Department of Internal Medicine Kaohsiung Medical University Hospital Taiwan; ^10^ College of Biological Science and Technology National Yang‐Ming Chiao Tung University Taiwan; ^11^ Institute of Biomedical Science National Sun Yat‐sen University Kaohsiung Taiwan

**Keywords:** adipokines, rheumatoid arthritis, sarcopenia

## Abstract

Large cohort studies have disclosed the association between obesity and rheumatoid arthritis (RA) risk. The sarcopenia prevalence in RA patients can be up to 31%. However, there is little information linking adipokines to sarcopenia in RA, so this study aimed to investigate whether adipokines were indeed involved in secondary sarcopenia in RA with a focus on non‐obese females. Sixty‐four female patients and 36 controls were included in this study. The serum adipokine levels (leptin and adiponectin) were determined by ELISA kits. The impacts of adipokines on muscle atrophy and potential autophagy were examined in mouse myoblasts, C2C12, upon treatment with recombinant leptin and adiponectin agonist (AdipoRan). Interestingly, serum adiponectin was significantly increased but the ratio of leptin/adiponectin was dramatically decreased in the RA patients with sarcopenia. After normalization by body mass, serum leptin was positively associated but adiponectin was negatively associated with muscle mass respectively, even after adjustment for fat mass. Treating C2C12 cells with leptin and AdipoRan inhibited proliferation of mature myotube respectively, as did treatment with the serum from RA patients. A combination of low leptin and high AdipoRan greatly decreased myogenin, but instead increased MAFbx and MuRF‐1 as well as increased Beclin 1, Atg5, and LC3β. Taken together, our study reveals that secondary sarcopenia of RA females may be an imbalance of RA‐related, but not obesity‐related, increase in adipokine production; additionally, the reduced leptin/adiponectin ratio could be a better indicator in monitoring sarcopenia in non‐obese RA females. Moreover, adipokine imbalance may promote muscle atrophy through inducing autophagy.

## 1 INTRODUCTION

Rheumatoid arthritis (RA) is a chronic inflammatory disease with autoimmune origin, characterized by synovial inflammation, cartilage damage, and bone erosion. The progression of RA is influenced by a combination of genetic and environmental factors, including hormonal changes and alcohol consumption.^1^ Notably, current research has identified a strong association between obesity and RA, impacting the risk of onset, subjective disease activities, and response to treatment.^2^ Several extensive cohort studies including EIRA (Epidemiological Investigation of Rheumatoid Arthritis), Nurses' Health Study (NHS), Danish National Birth Cohort (DNBC), European Prospective Investigation of Cancer (EPIC‐Norfolk), and Malmo Diet Cancer Study (MDCS) have also unveiled a significant correlation between obesity and RA.^2^, ^3^, ^4^, ^5^, ^6^, ^7^ A meta‐analysis further indicates that obese individuals have a relatively risk of 1.31 for developing RA, though some heterogeneity exists within the findings.^8^

Accumulating evidence has firmly established a correlation between adipokines and the pathogenesis of RA. Adipokines, a group of cytokines secreted by adipose tissue, play a crucial role in influencing both body composition and the immune system.^9^ This diverse group includes inflammatory mediators such as IL‐1 beta, IL‐6, and IL‐8, angiogenic factors like VEGF, and metabolic regulators including adiponectin, leptin, and TNF‐alpha.^10^ In RA patients, inflammatory cytokines like TNFα and IL‐1β induce higher metabolism and are associated with decreased fat‐free mass.^11^ Leptin levels are also elevated in RA patients, although they are lower in synovial fluid compared to plasma, suggesting local consumption of leptin within the joint cavity.^12^ It is important to note that there is heterogeneity in leptin levels among RA patients in previous studies.^13^ Additionally, baseline leptin levels have shown promise as predictors of RA activity and response to RA treatment within a six‐month timeframe.^14^ Adiponectin, another significant adipokine, plays a role in the pathophysiology of RA by inducing cytokines and matrix‐degrading enzymes in synovial fibroblasts.^15^ Cross‐sectional studies have demonstrated a positive association between adiponectin levels in RA patients and radiographic joint damage.^16^ An intricate involvement of adipokines in the inflammatory processes and joint damage seen in RA highlights their potential as both diagnostic markers and therapeutic targets for managing the disease.

Rheumatic diseases pose a significant risk for secondary sarcopenia.^17^ According to a recent meta‐analysis of 17 articles, the pooled prevalence of sarcopenia in RA patients is approximately 31%.^18^ The sarcopenia in RA was not associated with severity of RA, by DAS‐28 (disease activity score by 28 joints), but was associated with increased body mass index (BMI), increased fat mass, raised C reactive protein (CRP) and tumor necrosis factor‐α (TNF‐α).^19^ Sarcopenia is characterized by lower muscle mass/function and is associated with increased risks of falls, disability and mortality in the elderly.^20^ Long‐term studies indicate that, after 3 years of follow‐up, individuals with sarcopenia have a tripled mortality rate compared to those without, in the community.^21^ The prevalence of sarcopenia in the elderly in Taiwan ranges from 7.1% to 23.9%, as reported in various studies.^22^ Importantly, sarcopenia is a reversible condition. Interventions involving multiple components, including controlling underlying diseases, nutritional supplements and exercise, have shown promise in improving or reversing sarcopenia within a 3‐month timeframe.^22^ Various drugs targeting activin receptor, myostatin, MAS receptor, ryanodine receptor, vitamin D, angiotensin‐converting enzyme inhibitors, and anabolic agents such as ghrelin have been suggested as potential treatment options for sarcopenia. However, as of now, there is no approved medication specifically designated for sarcopenia treatment.^23^

Sarcopenia, typically associated with aging, can also be influenced by various risk factors, leading to what is termed secondary sarcopenia. Factors such as obesity, rheumatic diseases, and malnutrition contribute to this condition.^17^ Adipokines, including leptin, adiponectin, resistin, and apelin play crucial roles in sarcopenia.^24^ Although there is increasing evidence highlighting the impact of adipokines on sarcopenia, with only physical exercise being reported to reverse their effects,^25^ adipose tissue‐dependent metabolic effects such as oxidative stress, inflammation, and insulin resistance can independently lead to mitochondrial dysfunction, impaired insulin signaling and muscle atrophy.^26^ The intricate cross‐talk between myokines (muscle‐derived cytokines) and adipokines contributes significantly to the development of secondary sarcopenia.^25^

It is essential to recognize sarcopenia as a reversible precursor to disability and mortality, although it is often underdiagnosed and undertreated.^27^ Investigating the relationships between adipokines and sarcopenia will not only enhance our understanding of the disease processes but also pave the way for the development of targeted treatments for sarcopenia in individuals with RA. Further research into the mechanisms underlying the association of adipokines with RA and sarcopenia is therefore crucial, so the present study aimed to elucidate the role of adipokines in RA‐associated sarcopenia. Specifically, we investigated the association of adipokines with sarcopenia in non‐obese RA females, allowing us to discern the impact of adipokines on sarcopenia independent of obesity‐related factors.

## 2 MATERIALS AND METHODS

### 2.1 Patients and the diagnosis of RA, frailty, and sarcopenia

All study participants were recruited from the clinics of two hospitals, and their enrollment was contingent upon providing informed consent. The study protocol received approval from the Institutional Review Board of Kaohsiung Medical University Hospital (KMUIRB‐20170046). There were 64 RA patients and 36 controls included in the study. The patients' written informed consent forms were obtained. RA was diagnosed based on the 2010 ACR/EULAR (American College of Rheumatology/European League Against Rheumatism) Rheumatoid Arthritis Classification criteria.^28^ The clinical frailty scale (CFS) was used for frailty diagnosis. The CFS was the simplified form of frailty index and was verified.^29^ We routinely used the CFS in our hospital to find frail people and provide interventions. The frail group people in our study were rated as CFS 4–5 points (four points as very mildly and five points as mildly frail people) and without sarcopenia.^29^ Sarcopenia was diagnosed following the 2019 Asian Sarcopenia Working Group's diagnostic criteria.^20^ The cut‐off values of sarcopenia were as follows: (A) impaired physical performance: walking speed <1 m/s or five times chair stands ≧12 s, (B) grip strength: less than 28 kg in men or 18 kg in women, (C) appendicular skeletal muscle mass (ASM) <7.0 kg/m^2^ in men and <5.7 kg/m^2^ in women by bioelectrical impedance analysis.^20^ (A) + (C) or (B) + (C) was diagnosed as sarcopenia. The muscle mass and fat tissue mass were measured via bioelectrical impedance analysis (BIA) machine Inbody S10.^30^ The diagnoses of frailty and sarcopenia were not affected by the RA severity. Patient characteristics and laboratory data were obtained from the medical records.

### 2.2 RNA extraction of peripheral blood mononuclear cells

The PBMCs of 64 RA patients and 36 healthy controls were isolated using the Ficolle Paque (GE Healthcare, USA). Commercial kits (QIAmp RNA blood mini kit, Qiagen, Germany) were used to extract the RNA of the PBMCs respectively.

### 2.3 Enzyme‐linked immunosorbent assay for serum adipokines

Adipokine concentrations (adiponectin and leptin) were determined by sandwich enzyme‐linked immunosorbent assay (ELISA) kits. These commercial ELISA kits were purchased from Invetrogene (Invetrogene Systems). All experimental procedures were performed following the manufacturer's protocol.

### 2.4 Cells culture and treatment

The mouse skeletal muscle cell line, C2C12 (ATCCR CRL‐1772™) is used in this study. The base medium for C2C12 was ATCC‐formulated Dulbecco's Modified Eagle's Medium (DMEM). Fetal bovine serum (FBS) in the base medium was added to a final concentration of 10% to make the complete growth medium. The muscle cells were incubated in 5% carbon dioxide (CO_2_) and 37°C. After myoblast cells had reached 80% confluence, the cells were replaced with muscle differentiation medium [DMEM supplemented with 2% FBS] for 5 days, and then transformed to myotubes. To investigate the effects of patient serum on C2C12 myotube, the RA patient serum samples, containing adiponectin and leptin, were added onto C2C12 myotubes after the transformation trigger for 3 days, then after harvesting for an additional 48 h, the protein and mRNA were collected for western blot and real‐time qPCR respectively. AdipoRon was purchased from AdipoGen (San Diego, CA, USA), while mouse recombinant leptin was obtained from PeproTech (Rocky Hill, NJ, USA). After the initial differentiation for 3 days, the recombinant leptin (0–40 ng/mL) and adiponectin receptor agonist (AdipoRon, 0–40 M) were added onto C2C12 myotubes, then after harvesting for an additional 48 h, the protein and mRNA were collected for western blot and real‐time qPCR respectively.

### 2.5 XTT assay

After treatment, the cell proliferation rate was determined by the XTT colorimetric cell proliferation assay (Roche Molecular Biochemicals). Briefly, after treatment with patient serum, recombinant leptin or adiponectin receptor agonist (adipoRon) for 24–48 h, the culture medium in the 96‐well plate was removed and 100 μL of fresh culture medium and a pre‐formulated 50 μL XTT mixed reagent (XTT reagent:electronically coupled reagent = 50:1) were added, then the culture plate was incubated at 37°C for 4 h. Light absorbance values were read at wavelengths of 490 and 650 nm using an ELISA reader.

### 2.6 The quantitative PCR

After treatment, the total RNA was isolated by TRIzol reagent (1 mL; Invitrogen, Carlsbad, CA, USA). Briefly, TRIzol reagent was added to the treated cells and mixed well by vortex and then incubated at room temperature for 10 min to effectively denature proteins. 1‐Bromo3‐chloropropane (0.2 v/v) was then added and mixed by inverting the tubes for 15 s to separate aqueous and organic phases. Next, the samples were centrifuged at 12,000 rpm for 12 min at 4°C. All RNAs, including small RNAs, were in the aqueous phase, which was the top clear phase.

The cDNA was obtained after reverse transcription of mRNA extracted from the total RNA by the high production cDNA Archive Kit with the random primer (Applied Biosystems, USA). SYBR GREEN gene expression assay and QuantStudio™ 3 System (Life Technologies) were used to perform real‐time qPCR. β‐Actin were used as the endogenous control in this study. The PCR condition was as follows: (1) initial incubation at 50°C for 2 min, (2) enzyme activation step at 95°C for 10 min, and (3) 40 cycles of denaturation at 95°C for 15 s and annealing extension at 60°C for 1 min (the SYBR GREEN probe hybridization temperature is 60°C). The relative expression was calculated by the 2^−ΔCT^ method after being normalized to β‐actin expression. The following primers were used: MAFbx‐Forward 5′‐CTTTCAACAGACTGGACTTCTCGA‐3′, Reverse 5′‐CAGCTCCAACAGCCTTACTACGT‐3′; MuRF1: Forward 5′‐GCTGGTGGAAAACATCATTGACAT‐3′, Reverse 5′‐CATCGGGTGGCTGCCTTT‐3′; Myogenin: Forward 5′‐ATGGTGCCCAGTGAATGCAA‐3′, Reverse 5′‐ACCCAGCCTGACAGACAATC‐3′; β‐Actin: Forward 5′‐CCCAGCACAATGAAGATCAA‐3′, Reverse 5′‐ACATCTGCTGGAAGGTGGAC‐3′.

### 2.7 Western blot analysis

After treatment, the cells were also dissolved in the Triton X‐100 lysis buffer (10 mM Tris–HCl pH 7.4, 100 mM NaCl, 1 mM EDTA, 1 mM EGTA, 1 mM NaF, 2 mM Na_3_VO_4_, 1% Triton X‐100, 10% glycerol, 0.1% SDS, 0.5% deoxycholate, 1 mM PMSF, 1 mg/mL leupeptin, and 5 mL/mL aprotinin). The extracts were centrifuged at 13,000 rpm for 15 min at 4°C. The protein concentrations in the supernatant were determined by Bradford assay using the Bio‐Rad protein assay kit (Bio‐Rad). Equal amounts of total protein (~150 μg) were separated on 10% sodium dodecyl sulfate (SDS)–polyacrylamide gel electrophoresis (SDS PAGE) gels, then transferred to PVDF membranes using a Semi‐Dry Transfer Cell (Bio‐Rad). Next, 2 mL of 1× blocking buffer was added and samples were incubated at room temperature for 30 min to block membranes, followed by a further 2 h to incubate the membranes. They were then hybridized with primary antibody for 2 h at room temperature (RT), identified with a secondary antibody for 1 h at RT, and exposed to Kodak film. The following antibodies were used for immunoblotting: β‐actin, myogenin, MAFbx, MuRF1, and autophagy‐associated marker (Atg5, LC3 α/β, and Beclin 1) purchased from Santa Cruz Biotechnology (Dallas, TX, USA).

### 2.8 Statistical analysis

The *t*‐test, Spearman's correlation, one‐way ANOVA and post hoc analysis were used to compare the group differences, with a *p* value less than 0.05 considered statistically significant. The SPSS program was used for the above calculations.

## 3 RESULTS

### 3.1 The anthropometric measurements of those participants

We enrolled a total of 100 women in the study. The anthropometric measures and physical performances (handgrip strength and 6‐m walking time) are listed in Table 1. Subjects with frailty (CFS points 4–5) in the study had slower walking speed but did not meet the criteria of sarcopenia. Sarcopenic patients with RA had worse physical performance than sarcopenic people without RA, though the former group was younger than the latter. Frail patients in both groups had increased abdominal circumferences (more than 80 cm). Though the sarcopenia group's body mass indexes (BMI) were low in both groups, their abdominal circumferences were not statistically significantly lower than other groups.

**TABLE 1:** Physical characteristics and bioimpedance parameters.

Numbers (total *N* = 100)	People without autoimmune diseases			RA patients		
	Control (*N* = 6)	Frailty (*N* = 12)	Sarcopenia (*N* = 18)	RA (*N* = 35)	RA with frailty (*N* = 16)	RA with sarcopenia (*N* = 13)
General characteristics						
Age	70.8 ± 8.3	76.9 ± 8.4	75.7 ± 10.1	67.9 ± 6.0	72.6 ± 9.0^$^	70.8 ± 6.9
Body weight (kg)	57.8 ± 11.1	57.2 ± 7.7	51.2 ± 9.2	57.7 ± 11.9	59.0 ± 9.2	47.0 ± 9.4*, ^$$^, ^##^
Body length (cm)	152.4 ± 5.4	151.1 ± 5.6	151.4 ± 4.8	155.7 ± 6.1	153.2 ± 5.4	152.1 ± 5.7
Body mass index (kg/m^2^)	24.9 ± 6.0	25.0 ± 3.0	22.3 ± 4.0	23.9 ± 5.1	25.2 ± 3.1	20.2 ± 3.7*, ^$$^, ^##^
Waist (cm)	73.0 ± 7.9	87.1 ± 9.8*	85.2 ± 13.3	83.5 ± 13.0	88.6 ± 14.9	78.4 ± 11.7
Calf circumference						
Right leg (cm)	29.8 ± 2.5	32.0 ± 3.3	28.2 ± 3.2^#^	32.2 ± 3.7	29.7 ± 3.4	27.5 ± 2.4^##^
Left leg (cm)	30.5 ± 2.2	32.3 ± 3.1	28.2 ± 3.3^##^	31.9 ± 3.7*	29.8 ± 3.2	27.4 ± 2.0*, ^##^
Bioimpedance analysis (BIA)						
Skeletal muscle mass (kg/m^2^)	6.4 ± 0.3	6.5 ± 0.6	5.1 ± 0.5**, ^##^	6.8 ± 0.8	6.6 ± 0.6	5.0 ± 0.7**, ^$$^, ^##^
Handgrip strength (kg)	17.6 ± 2.0	16.6 ± 4.3^##^	11.5 ± 3.3**, ^##^	16.2 ± 5.0	11.3 ± 4.8*, ^$$^	9.7 ± 4.1**, ^$$^
Six meters walking time (s)	6.6 ± 0.2	12.7 ± 8.7	16.6 ± 9.4	5.8 ± 0.8	9.7 ± 3.1^$$^	10.1 ± 6.9^$$^

*Note*: Values are means ± SD.

Abbreviations: muscle mass: appendicular mass, measured by bioimpedance analysis; RA, rheumatoid arthritis.

* *p* < 0.05 versus control group.

** *p* < 0.01 versus control group.

^$^ *p* < 0.05 versus RA group.

^$$^ *p* < 0.01 versus RA group.

^#^ *p* < 0.05 versus RA/frailty group.

^##^ *p* < 0.01 versus RA/frailty group.

### 3.2 The leptin/adiponectin levels were significantly lower in the RA patients with sarcopenia

**FIGURE 1:**
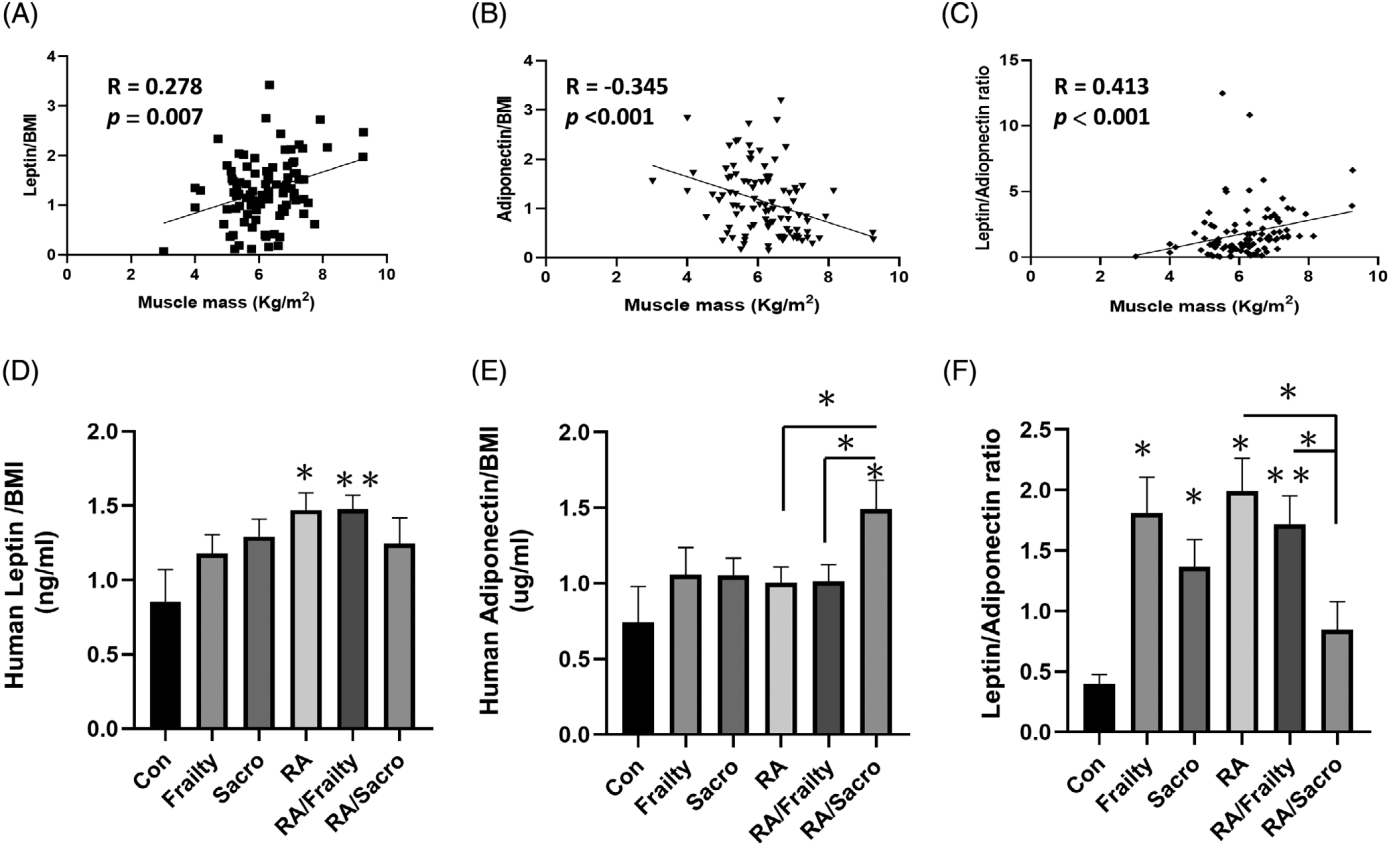
The ratio of leptin and adiponectin was correlated with muscle mass and decreased in RA patients with sarcopenia. After being normalized by body mass index (BMI), (A) leptin level was positively correlated with muscle mass. (B) Adiponectin level was negatively correlated with muscle mass. (C) The leptin/adiponectin ratio was correlated positively with muscle mass. (D) The leptin/BMI level was raised in the RA patients but decreased in the RA patients with sarcopenia. (E) The adiponectin/BMI was raised in those RA patients with sarcopenia. (F) The leptin/adiponectin ratio decreased in RA patients with frailty and further in those with sarcopenia. *p < 0.05 compared with the control group; **p < 0.01 compared with the control group; BMI, body mass index; con, control; RA, rheumatoid arthritis without frailty or sarcopenia; RA/Frailty, RA patients with frailty but no sarcopenia; RA/Sacro, RA patients with sarcopenia; sarco, sarcopenia.

The leptin level was correlated with the BMI.^31^ We normalized the serum leptin and adiponectin level with BMI to avoid interference. With data normalized by BMI, a negative correlation of adiponectin but a positive correlation of leptin to muscle mass were evident respectively (Figure 1A,B). Furthermore, the leptin/adiponectin ratio was positively associated with muscle mass (Figure 1C). The leptin/BMI level exponentially increased in the control group with frailty and further in those with sarcopenia, while the leptin/BMI level in the RA patients without frailty nor sarcopenia, was higher than those sarcopenia patients in the control group (Figure 1D). The leptin/BMI level in RA patients with sarcopenia was lower when compared with those RA patients without sarcopenia. The adiponectin/BMI level increased significantly only in the RA patients with sarcopenia and was significantly higher than the RA patient with and without frailty (Figure 1E). Intriguingly noted, the leptin/adiponectin ratio was lowest in the RA patients with sarcopenia than in the RA patients without and those with frailty (Figure 1F).

### 3.3 The leptin and adiponectin levels, normalized by body fat mass, were significantly correlated with muscle mass

**FIGURE 2:**
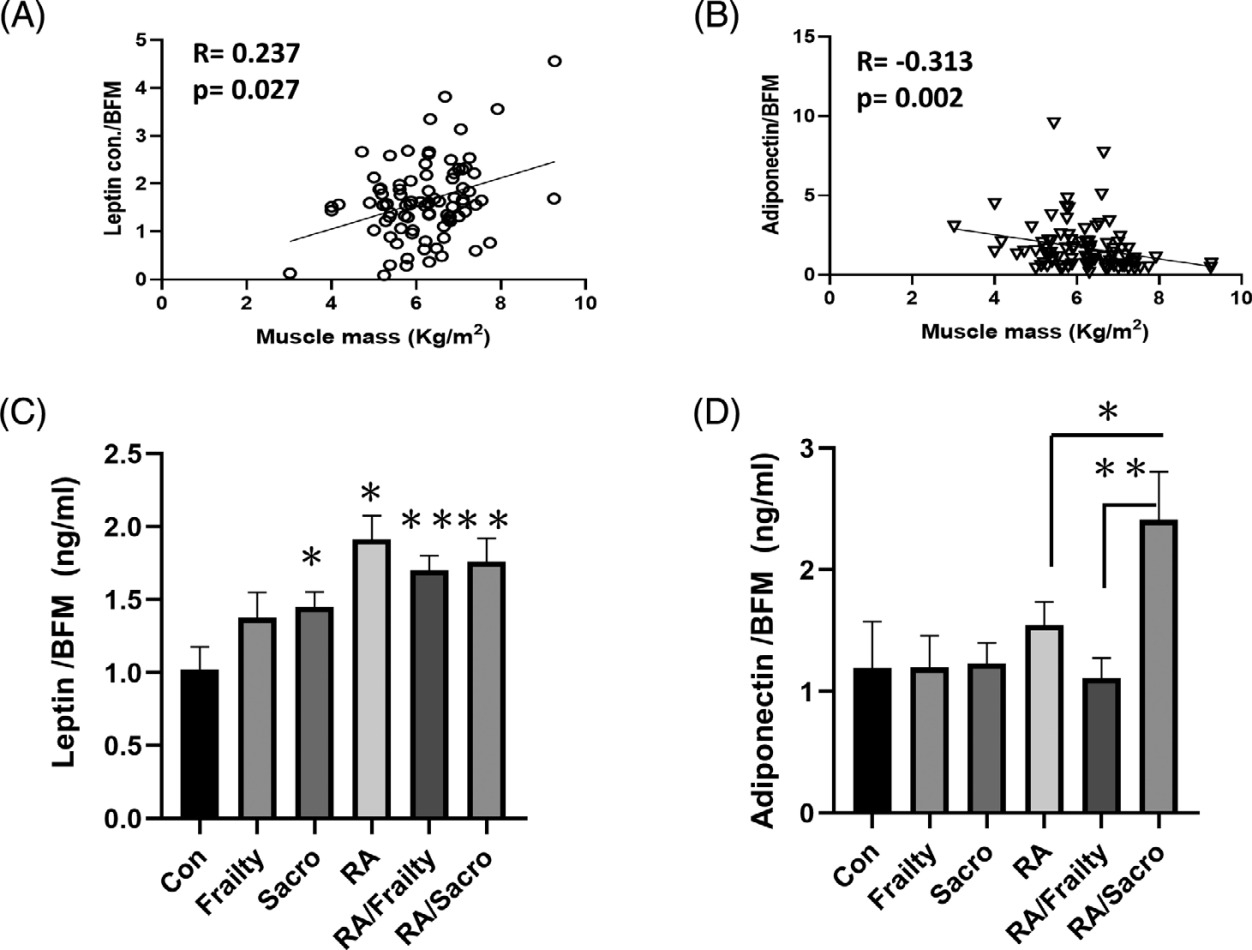
The leptin adiponectin levels normalized by body fat mass correlated with muscle mass. (A) After being normalized by body fat mass (BFM), leptin level was positively correlated with muscle mass. (B) The adiponectin, normalized by BFM, was negatively correlated with muscle mass. (C) The leptin level, normalized by body fat mass, was higher in the RA patients but lower in the RA patients with sarcopenia. (D) The adiponectin level, normalized by body fat mass, was higher significantly in those RA patients with sarcopenia. *p < 0.05; **p < 0.01 compared with the control group; con, control; RA, rheumatoid arthritis; RA/Frailty, RA patients with frailty but no sarcopenia; RA/Sacro, RA patients with sarcopenia; sarco, sarcopenia.

Considering that the leptin level is associated with the body fat mass (BFM).^32^ We normalized the serum leptin and adiponectin levels by BFM. A positive correlation of leptin with BFM and a negative association of adiponectin with BFM were found (Figure 2A,B). The leptin/BFM was increased in all RA patient groups and in those controls with sarcopenia (Figure 2C). Adiponectin/BFM showed a different pattern where it was the highest and significantly higher in RA with sarcopenia than in the other two RA patient groups (Figure 2D).

### 3.4 The correlation of adipokines with muscle mass changed in the subgroup analysis

**FIGURE 3:**
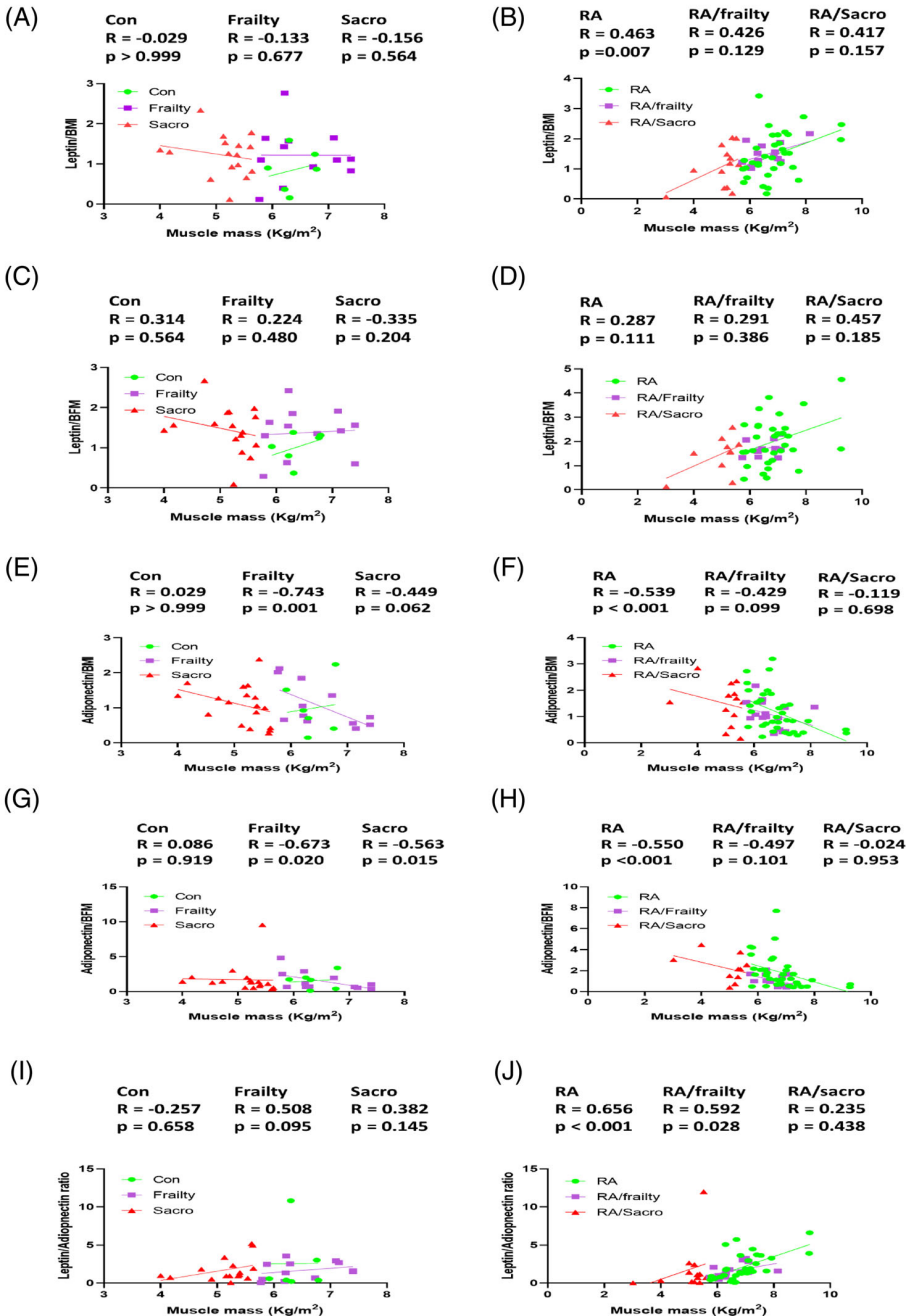
The trend of leptin and adiponectin changes in the subgroups was evident in the RA patients. The association between leptin/BMI or leptin/BFM and muscle mass were insignificant in three subgroups of people without RA (A and C). The leptin/BMI and leptin/BFM levels were raised with muscle mass in the three subgroups of RA patients (B and D). The adiponectin/BMI and adiponectin/BFM decreased with muscle in frail and sarcopenic subgroups in the people without rheumatoid arthritis (E and G). Adiponectin/BMI and adiponectin/BFM were negatively correlated with muscle in RA patients (F and H). The association of leptin/adiponectin with muscle mass was not statistically significant in subgroups of control people (I). The muscle mass was positively correlated with muscle mass in RA patients (J). BFM, body fat mass; BMI, body mass index; con, control; RA, rheumatoid arthritis without frailty and sarcopenia; RA/Frailty, RA patients with frailty but no sarcopenia; RA/Sacro, RA patients with sarcopenia; sarco, sarcopenia.

To understand the adipokine changes in the six subgroups, we checked their correlations between adipokines normalized by BMI or BFM, and muscle mass (Figure 3). The small sample size in each subgroup made it difficult to reach statistical significance; however, the trend of adipokine changes in subgroups could be seen by the slope lines of the dot plots. The changes in the leptin correlations were more evident and consistent in subgroups of RA patients than in the controls, where a significantly positive correlation of leptin/BMI with muscle was seen in only the RA patients (Figure 3A–D) while negative correlations of adiponectin normalized to BMI and BFM with muscle were also found in only the RA patients (Figure 3E–H). A significantly positive correlation of leptin/adiponectin ratio with muscle was demonstrated in the RA patients and those with frailty (Figure 3I,J). The slope of leptin was diagonally upward, whereas the adiponectin was diagonally downward; meanwhile, in the control group, the changes in adiponectin normalized by BMI and BFM of the frail and sarcopenia groups were noticeable (Figure 3E,G).

### 3.5 Treating the myotubes with adipokines and the RA patients' serum impaired the proliferation of myotube cells

**FIGURE 4:**
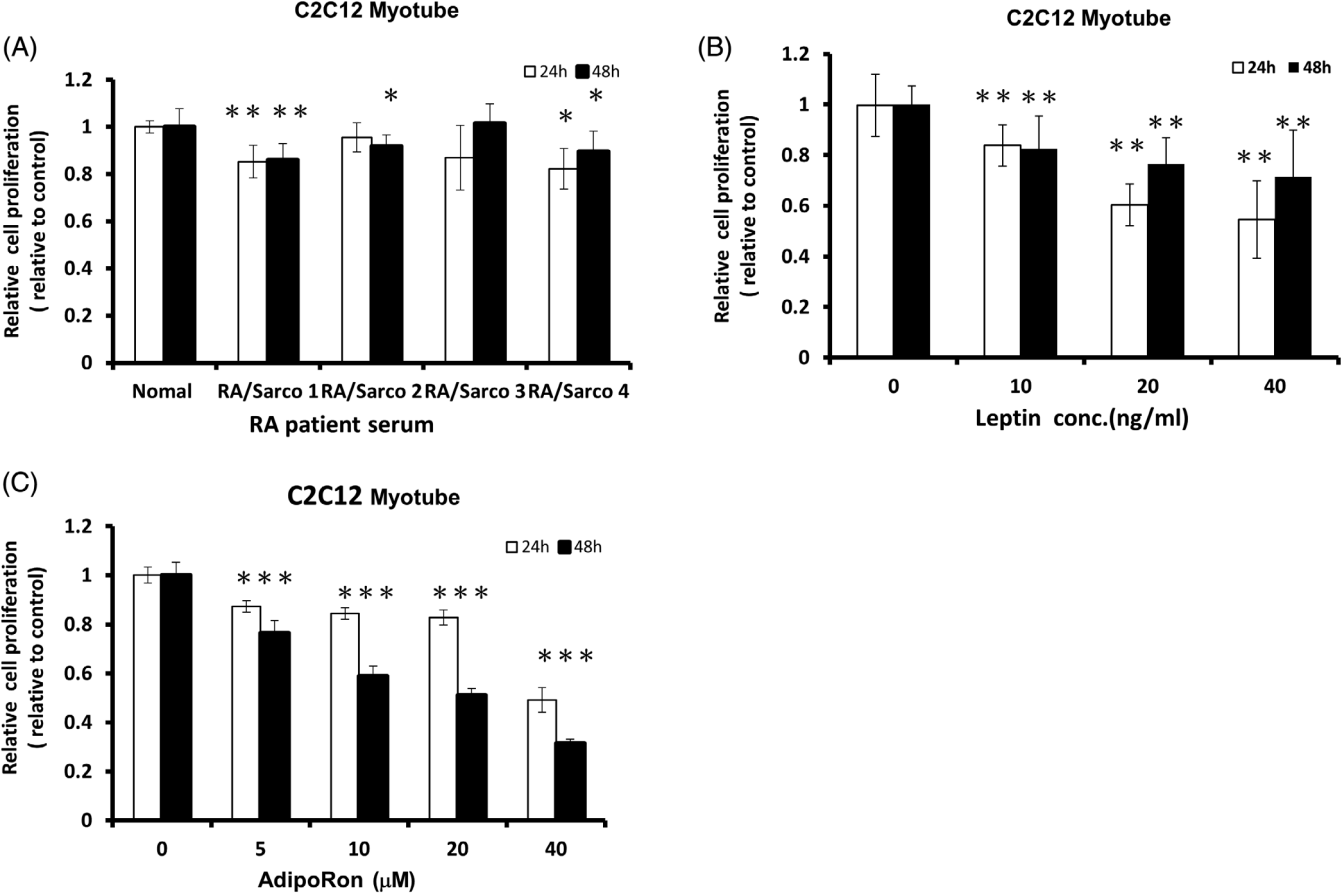
Leptin and adiponectin decreased the proliferation of myotubes in vitro. (A) The proliferation of myotubes was suppressed by the serum of RA patients with sarcopenia. (B) The leptin suppressed the myotubes proliferation. (C) The dose effect of adiponectin agonist (AdipoRon) on suppressing mice myotubes was noticeable. *p < 0.05; **p < 0.01 compared with the control group.

After treating the myotubes with the serum of RA patients with sarcopenia, the proliferation of C2C12 myotube was generally suppressed (Figure 4A). The RA patients' serum was diluted in cell cultures for myotubes' survival; thus, the effect of serum might be attenuated. We further treated the myotubes with recombinant leptin and adiponectin agonist (adipoRon). The proliferation of myotube cells was significantly decreased by recombinant leptin and adipoRon in a dose‐ and time‐dependent manner (Figure 4B,C). Although the dose effect was more evident in adipoRon than in leptin. The myotube proliferation was suppressed by recombinant leptin in the first 24 h, but the effect subsided partially after 48 h.

### 3.6 The combined leptin and adiponectin showed a dose‐dependent effect on muscle degradation

**FIGURE 5:**
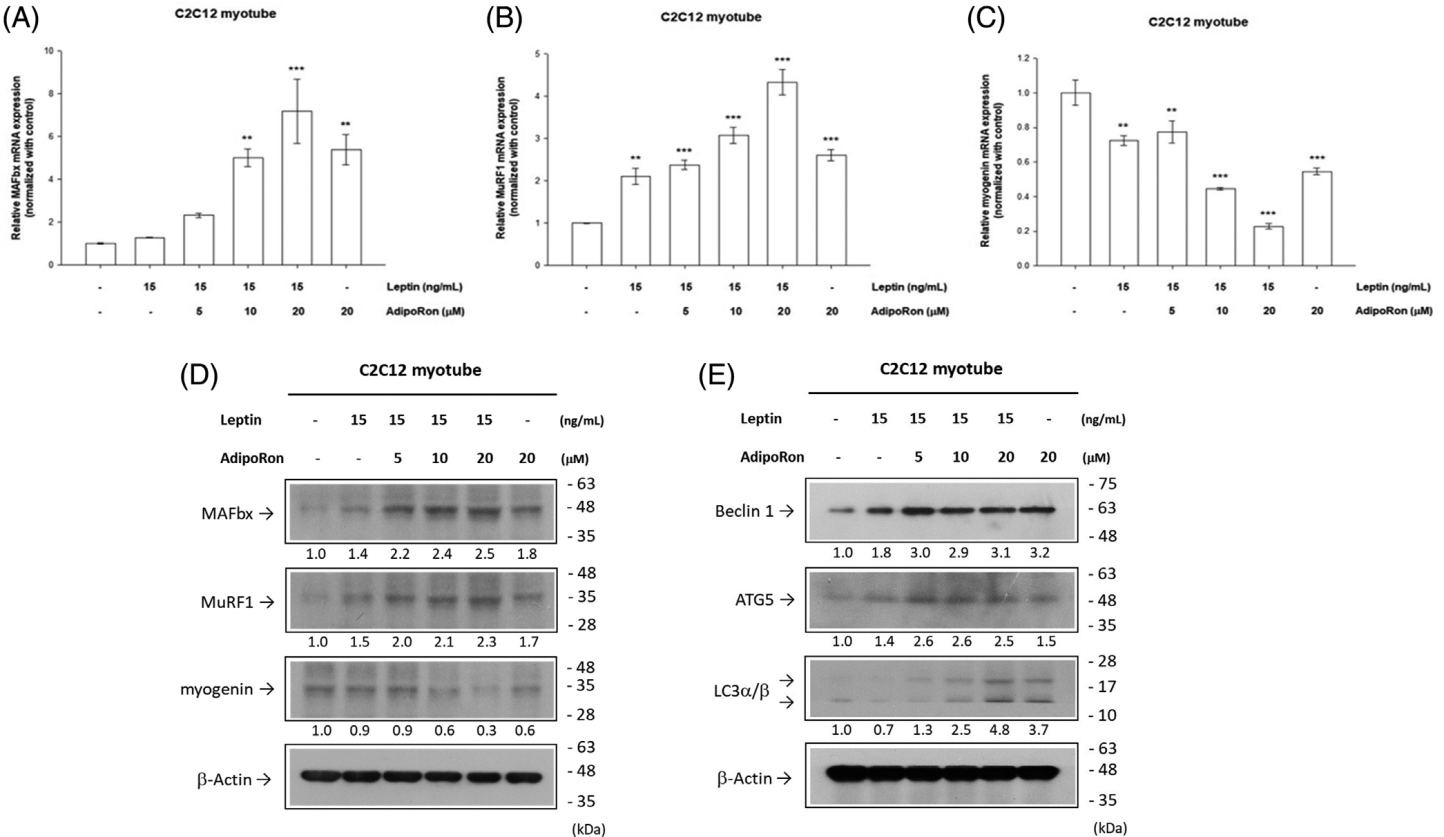
Adipokines affect muscle via autophagy pathway. Treating the myotubes, transformed from C2C12 myoblast lines, with leptin/adiponectin agonist increased muscle degradation. The dose‐dependent effect was pronounced. Treating cells with leptin/adiponectin enhanced the mRNA expressions of (A) MAFbx and (B) MURF1 and attenuated those of (C) myogenin. (D) The western blot result confirmed these changes in protein level. (E) The Beclin 1, ATG5 and LC3α/β, markers of autophagy, increased in the leptin/adiponectin co‐treated muscle cells. ATG5, autophagy related 5; LC3α/β, light chain 3α/β; MAFbx, muscle atrophy F‐box; MuRF1, muscle RING‐finger protein‐1.

The effects of leptin and adiponectin on muscle cells were contrary; however, they were both raised in RA patients.^15^ We treated the C2C12 with fixed concentrations of recombinant leptin and different dosages of adipoRon and found adipoRon could promote muscle cell degradation in a dose‐dependent manner. The mRNA levels of the muscle degradation markers (MuRF‐1 and MAFbX) were increased, but those of the proliferation marker, myogenin (MyoG), were decreased after treatments (Figure 5A–C). The highest dose of adipoRon and fixed concentration of recombinant leptin had the most evident effects, and similar patterns were also seen in protein levels (Figure 5D). Meanwhile, the autophagy biomarker including Beclin 1, autophagy related 5 (ATG5), and light chain 3α/β (LC3α/β) were increased (Figure 5E). Thus, increased adiponectin plus higher leptin might promote muscle degradation and autophagy.

## 4 DISCUSSION

The present study focused on the association between leptin, adiponectin, BMI, BFM, and muscle mass in non‐obese participants, particularly in the context of RA. This study included women only because leptin could be upregulated by the ovarian sex hormone.^33^ Leptin was associated with BMI in RA patients, suggesting a relationship between body weight and this adipokine.^13^ Both were done to avoid any possible interferences, and the association between muscle mass and the ratio of leptin/adiponectin was apparent after adjusting for BMI. Leptin is active at the central nervous system level to suppress food intake needs and stimulate energy expenditure.^34^ The raised leptin impact on muscle mass is controversial. In sarcopenic obesity, elevated leptin did not show a positive effect on muscle mass, called leptin resistance.^24^ In our non‐obese participants, the leptin was positively associated with muscle mass. Most reports have indicated that adiponectin was protective for muscle cells^24^; however, some reports indicate a negative correlation between the circulation level of adiponectin and muscle mass and physical functioning in older people.^35^, ^36^ In our study, we provided evidence of the negative association between adiponectin and muscle mass and also confirmed the effect by the cell model. Considering the co‐existence and contrary effects of leptin and adiponectin in the muscle mass, it is inappropriate to use the value of a single adipokine alone to determine adipose tissue's effect on muscle. The leptin/adiponectin ratio might be a better indicator to decide the actual effect of adipokines on muscle mass, at least in RA patients.

Leptin and adiponectin are mainly produced by adipose tissue.^33^ In Figure 2, we adjusted the leptin and adiponectin levels by BFM, and found that RA patients' leptin and adiponectin levels were higher in the same amount of adipose tissue than those without RA. This was compatible with their roles in inflammation.^16^ Normalized adiponectin concentrations by BFM correlated positively to the degree of bone destruction by Steinbrocker classification in RA patients.^37^ Even when adjusted by BFM, the association of leptin and adiponectin with muscle mass did not change.

We treated the diluted RA patients' serum on the muscle cells in vitro, with the serum being diluted so that the muscle cells could survive. Muscle proliferation was suppressed at varying levels, with lower concentrations of adipokines, while treating muscle cells with recombinant leptin or adiponectin agonist suppressed muscle proliferation. Muscle wasting is a common characteristic of sarcopenia, mainly resulting from decreased protein synthesis and increased breakdown.^38^ Mitochondrial and nuclear damage, decreased muscle‐specific PGC‐1α, apoptosis pathway, MAFbx and MuRF‐1‐mediated ubiquitination, and TNF‐α and INF‐γ‐induced inflammation all involve the mechanism of the development of sarcopenia.^39^ In our study, the muscle degradation markers (MAFbx and MuRF‐1) were increased, and the myogenesis marker (myogenin) was decreased after treating leptin/adiponectin at both mRNA and protein levels. The markers of the autophagy pathway (Beclin 1, ATG5, and LC3 alpha/beta) increased when treating muscle cells with adipokines, showing that the adipokines induced autophagy and followed muscle atrophy.

To sum up, the adipokines may be not only involved in the inflammatory progression but also the sarcopenia development in RA patients. The synergistic crosstalk between leptin and adiponectin might better indicate adipokines' effect on the muscle rather than a single one. Early detection and intervention of sarcopenia is necessary to delay or prevent disabilities.^40^ The RA patients are at high risk of disability for bone and joint destruction, and the high prevalence of sarcopenia in RA patients warrants more attention when under treatment. Our study underscores the multifaceted impact of adipokines in RA, extending beyond inflammation to include musculoskeletal aspects such as sarcopenia.

## CONFLICT OF INTEREST STATEMENT

The authors declare no conflict of interest.
